# Comprehensive analysis and molecular map of Hippo signaling pathway in lower grade glioma: the perspective toward immune microenvironment and prognosis

**DOI:** 10.3389/fonc.2023.1198414

**Published:** 2023-05-12

**Authors:** Yu-Duo Guo, Jie Sun, Chao Zhao, Le Han, Chun-Jiang Yu, Hong-Wei Zhang

**Affiliations:** ^1^ Department of Neurosurgery, Sanbo Brain Hospital, Capital Medical University, Beijing, China; ^2^ Rehabilitation Department of Integrated Chinese and Western Medicine, Beijing Xiaotangshan Hospital, Beijing, China; ^3^ Chinese Academy of Sciences (CAS) Key Laboratory of Infection and Immunity, Institute of Biophysics, Chinese Academy of Sciences, Beijing, China

**Keywords:** LGG, Hippo signaling pathway, machine learning, immune microenvironment, prognosis

## Abstract

**Background:**

The activation of YAP/TAZ transcriptional co-activators, downstream effectors of the Hippo/YAP pathway, is commonly observed in human cancers, promoting tumor growth and invasion. The aim of this study was to use machine learning models and molecular map based on the Hippo/YAP pathway to explore the prognosis, immune microenvironment and therapeutic regimen of patients with lower grade glioma (LGG).

**Methods:**

SW1783 and SW1088 cell lines were used as *in vitro* models for LGG, and the cell viability of the XMU-MP-1 (a small molecule inhibitor of the Hippo signaling pathway) treated group was evaluated using a Cell Counting Kit-8 (CCK-8). Univariate Cox analysis on 19 Hippo/YAP pathway related genes (HPRGs) was performed to identify 16 HPRGs that exhibited significant prognostic value in meta cohort. Consensus clustering algorithm was used to classify the meta cohort into three molecular subtypes associated with Hippo/YAP Pathway activation profiles. The Hippo/YAP pathway’s potential for guiding therapeutic interventions was also investigated by evaluating the efficacy of small molecule inhibitors. Finally, a composite machine learning models was used to predict individual patients’ survival risk profiles and the Hippo/YAP pathway status.

**Results:**

The findings showed that XMU-MP-1 significantly enhanced the proliferation of LGG cells. Different Hippo/YAP Pathway activation profiles were associated with different prognostic and clinical features. The immune scores of subtype B were dominated by MDSC and Treg cells, which are known to have immunosuppressive effects. Gene Set Variation Analysis (GSVA) indicated that subtypes B with a poor prognosis exhibited decreased propanoate metabolic activity and suppressed Hippo pathway signaling. Subtype B had the lowest IC50 value, indicating sensitivity to drugs that target the Hippo/YAP pathway. Finally, the random forest tree model predicted the Hippo/YAP pathway status in patients with different survival risk profiles.

**Conclusions:**

This study demonstrates the significance of the Hippo/YAP pathway in predicting the prognosis of patients with LGG. The different Hippo/YAP Pathway activation profiles associated with different prognostic and clinical features suggest the potential for personalized treatments.

## Introduction

Lower grade gliomas (LGG) are a group of slow-growing brain tumors that arise from glial cells. LGG are defined as World Health Organization (WHO) grade II or III tumors, which means that they are considered less aggressive than high-grade gliomas (WHO grade IV). Although these tumors are considered low grade, they can still be difficult to treat and have a significant impact on patient quality of life. Surgery remains the primary treatment option for LGGs, with the goal of achieving maximal safe resection while preserving neurologic function (1). However, the location and size of the tumor, as well as its proximity to critical brain structures, can make complete resection challenging (2). Despite these advances, treatment for LGGs remains a major challenge. The slow-growing nature of these tumors means that patients may experience significant disease progression before treatment is initiated. Additionally, the potential for recurrence is high, even after aggressive treatment.

Recent studies have shown that the Hippo signaling pathway is involved in the development and progression of LGG. The classical Hippo pathway plays a crucial role in regulating organ size and tissue homeostasis, and its dysregulation has been implicated in several cancers, including LGG (3). The Hippo pathway plays a central role in regulating organ size and maintaining dynamic tissue balance. Aberrant activation or inactivation of Hippo signaling components, such as YAP and TAZ, has been reported in LGG, and is associated with tumor growth, invasion, and prognosis (4). However, the precise mechanisms by which the Hippo pathway contributes to LGG development and progression, as well as its relationship with the immune microenvironment, remain to be fully elucidated. MST1/2 plays a selective role in the homeostasis and priming of CD8^+^ T cells, mediated by dendritic cells (5). Understanding the molecular map of the Hippo signaling pathway in LGG and its interaction with the immune microenvironment could provide new insights into LGG pathogenesis and identify potential therapeutic targets for this disease.

Hence, this study aimed to explore the prognosis, immune microenvironment, and therapeutic regimen of patients with LGG using a machine learning model, such as random forest (RSF), gradient boosting machine (GBM), survival support vector machine (Survival-SVM), supervised principal components (SuperPC), ridge regression, partial least squares regression for Cox (plsRcox), CoxBoost, Stepwise Cox (StepCox), and elastic network (Enet), etc. Results showed that different Hippo/YAP Pathway activation profiles were associated with different prognostic and clinical features. Subtype B had the worst prognosis, subtype C had the best prognosis, and subtype A was in between. Importantly, Subtype B had the lowest IC50 value, indicating sensitivity to targeted drugs based on the Hippo/YAP pathway. Our approach aimed to identify a comprehensive molecular signature associated with Hippo signaling in LGG, which could provide a more complete understanding of the biological mechanisms involved in LGG development and progression. Our study’s molecular map could help improve LGG diagnosis and prognosis, as well as identifying potential targets for LGG treatment.

## Materials and methods

### Cell culture

The SW1783 and SW1088 cell lines served as *in vitro* models for Low Grade Glioma (LGG), based on previous research (6), due to their shared molecular and genetic characteristics with human LGG. The cell lines were provided by Procell, Wuhan, China. The SW1783 and SW1088 cells were cultured using Gibco BRL’s Dulbecco’s Modified Eagle Medium (DEME) containing 10% fetal bovine serum. We dissolved the XMU-MP-1 (Catalog number S8334, Selleck Chemicals) in Dimethyl sulfoxide (DMSO) and added it to the medium at final concentrations of 0.1% or 0.2% (7). XMU-MP-1 is a small-molecule inhibitor of the Hippo signaling pathway.

#### Cell counting kit-8

A 96-well plate was used to culture SW1783 and SW1088 cells, with 2500 cells per well, and varying final concentrations of XMU-MP-1. The plate was incubated at 37°C for two hours, after which absorbance was measured at 450 nm using 10 μL of the CCK-8 reagent (Dojindo Molecular Technologies, Japan). Assays were performed every 24 h.

#### Edu and colony formation

To perform the EdU assay, 2×10^4^ treated cells per well were seeded into plate and allowed to attach before conducting the assay following the manufacturer’s recommended protocol (Ribobio, China) for 5-Ethynyl-2’-deoxyuridine (EdU) incorporation. To initiate the colony formation experiment, we seeded 1×10^3^ cells per well into a 6-well plate and incubated them for 14 days. The cells were washed twice with PBS before fixing them in 4% paraformaldehyde for 15 minutes, followed by staining with Crystal violet (Solarbio, China).

#### Pre-processing of raw data

The present study included transcriptomic data and clinical information from 481 samples obtained from the The Cancer Genome Atlas (TCGA) database, in accordance with previously established inclusion and exclusion criteria (8). Furthermore, transcriptomic data and clinical information from the Chinese Glioma Genome Altas (CGGA)-693 project (332 patients) and the CGGA325 project (162 patients) were downloaded from the CGGA database using the same criteria. Notably, all data analyzed in this study were obtained from RNA-Seq assays, with no duplicate sequencing samples, and exclusively from WHO grade II-III tumors. The data mentioned above were subjected to log normalization via TPM transformation, followed by batch effect correction using the Combat function within the “sva” R package (9). A total of 19 Hippo pathway-related genes (HPRGs) were obtained from the previous references (10), because it has been previously reported to be related to the Hippo pathway.

After hematoxylin and eosin (H&E) staining, the presence of inflammatory cells and other cells can be observed due to the distinctive characteristics of inflammatory cells, which typically have large nuclei and high nucleoplasm ratios. Specifically, the nuclei of inflammatory cells stain a distinct blue color with hematoxylin, while the cytoplasm is stained with eosin in varying shades of pink to peachy red. Furthermore, the intracytoplasmic eosinophilic granules appear bright red and highly reflective. Moreover, representative H&E-stained sections in different subtypes were downloaded from the TCGA database in svs format.These samples were obtained from the GDC Data Portal (https://portal.gdc.cancer.gov/). Due to the general treatment of H&E-stained section staining in TCGA database, we can only determine whether it is a high-inflammatory through the local area.

#### Consensus clustering analysis

We utilized the expression levels of the 19 HPRGs to establish the optimal number of clusters via unsupervised consensus clustering analysis, employing the “ConsensusClusterPlus” package. The effectiveness of this clustering approach was then evaluated through principal component analysis (PCA). The following was the parameter settings: reps=100, pItem=0.8, distance=“euclidean”, and clusterAlg=“km” (11, 12). By “clusters,” we mean groups of patients with similar gene expression patterns. These clusters were identified using unsupervised clustering analysis.

#### Enrichment analysis

The “limma” package was utilized to identify genes with significant expression changes, with a cut-off of an absolute log-fold change greater than 0.5 and a adj.p-value less than 0.05 (13). The “clusterProfiler” R package was applied to further annotate common differentially expressed genes (DEGs) (14), using a p-value and false discovery rate (FDR) q-value threshold of less than 0.05. To investigate differences in biological pathways between subtypes, gene set variation analysis (GSVA) was performed using the KEGG gene set, with an FDR threshold of less than 0.05 (15).

#### Machine learning-derived risk score

We implemented a previously established workflow to develop a consensus prognosis model for LGG patients (16). In brief, we combined 101 machine learning algorithms, such as random forest (RSF) based on ‘randomForestSRC’ package, gradient boosting machine (GBM) based on ‘gbm’ package, and survival support vector machine (Survival-SVM) based on ‘e1071’ package, among others, based on their features. We selected models with variable filtering capabilities as antecedent models. Using the workflow, we generated signatures from all Hippo pathway-related genes in an expression file, employing the TCGA-LGG cohort and 101 combinations. Subsequently, we calculated risk scores based on these signatures in the TCGA-LGG and CGGA cohorts. We assessed the mean C-index of the three cohorts and selected the optimal consensus prognostic model.

#### Immune microenvironment analysis

The immune microenvironment was assessed using the Estimation of STromal and Immune cells in MAlignant Tumor tissues using Expression data (ESTIMATE) algorithm, which provided an overall measure of the immune status. Additionally, we utilized the ssGSEA algorithm, following references (17), to determine immune cell scores and to evaluate the distribution of specific immune cell types.

#### Drug sensitivity analysis

The “pRRophetic” package utilizes expression profiles of cell lines from Cancer Cell Line Encyclopedia (CCLE) database and their corresponding IC50 data as primary inputs (18). A predictive model is constructed using ridge regression and applied to predict the chemotherapeutic response in bulk RNA-seq data from clinical samples. We selected representative drugs targeting the Hippo pathway, including WZ.4003, FG-3019, XAV.939, verteporfin, and XMU-MP-1.

#### Statistical analysis

All statistical analyses were conducted using R software (version 4.1.2). Detailed descriptions of the statistical methods are provided in previous sections and references (19, 20). Statistical significance was set at P < 0.05.

## Results

### Hippo/YAP pathway influences proliferation of LGG cells

**Figure 1:**
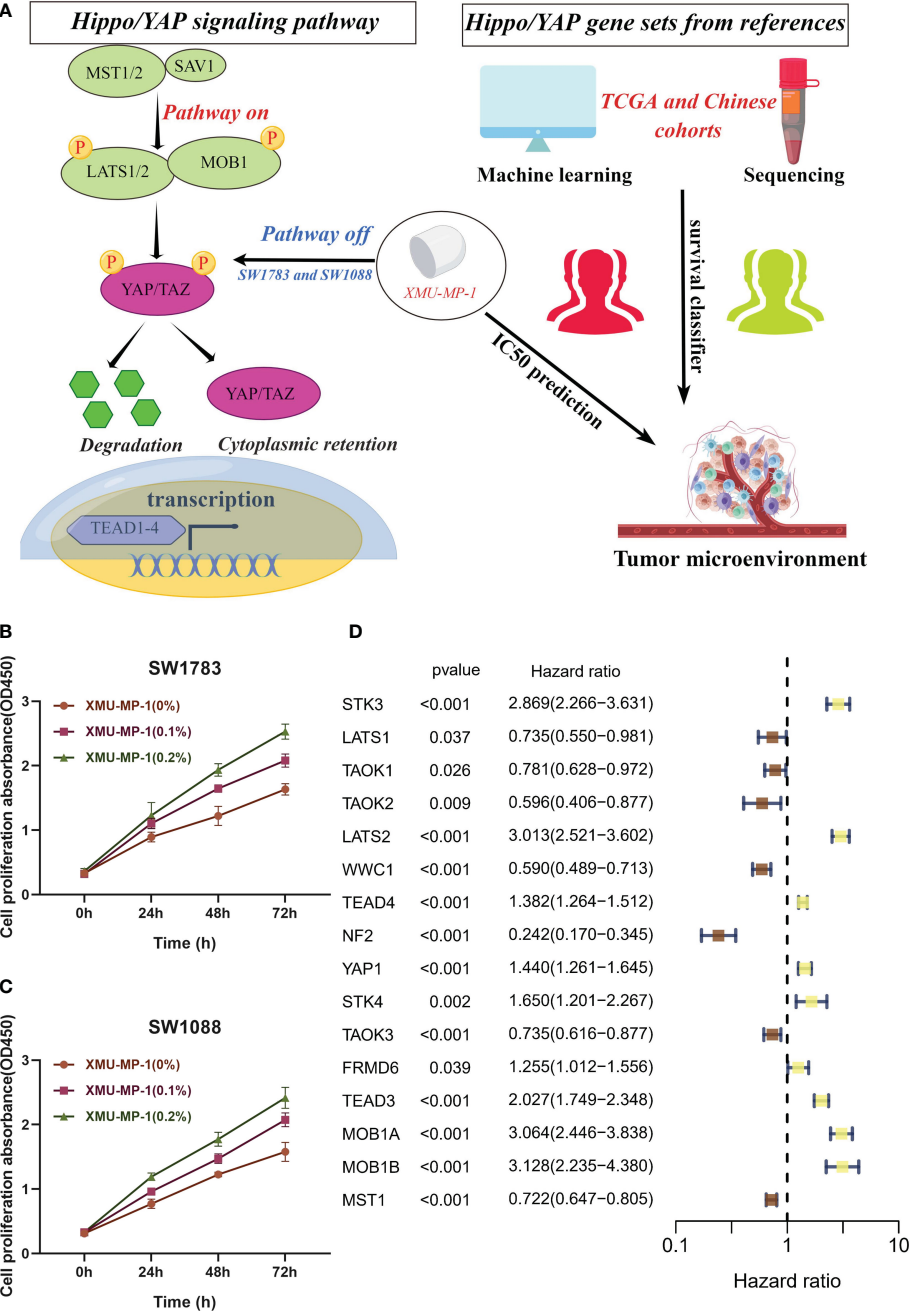
*In vitro* assays confirm the role of Hippo/YAP pathway in promoting proliferation of LGG cells. **(A)** Flowchart depicting the study design. **(B, C)** Cell viability assays of SW1783 and SW1088 cells treated with XMU-MP-1 reveal a dose-dependent increase in cell proliferation. **(D)** Univariate Cox analysis identifies 16 Hippo pathway-related genes (HPRGs) that exhibit significant prognostic value in the meta-cohort.

In numerous human cancers, the YAP/TAZ transcriptional co-activators, which are downstream effectors of the Hippo/YAP pathway, have been observed to be abnormally activated, resulting in the promotion of tumor growth and invasion (21). Conversely, the core kinase cassette of the Hippo pathway, which comprises MST1/2 and LATS1/2, has demonstrated tumor suppressive effects in several cancer types (3). We employed the gene set of the Hippo/YAP signaling pathway to develop a machine learning model to explore the prognosis of patients with LGG and forecast the IC50 of drugs targeting the Hippo/YAP signaling pathway, as illustrated in **Figure 1A**. XMU-MP-1 is a selective small molecule inhibitor of the Hippo/YAP pathway, which has been shown to specifically target and inhibit the activity of the downstream effector protein YAP (22). Based on previous research, we utilized SW1783 and SW1088 cell lines as *in vitro* models for LGG (10). These cell lines have been widely used in previous studies as they share similar molecular and genetic characteristics with human LGG. The cell viability of SW1783 (**Figure 1B**) and SW1088 (**Figure 1C**) was evaluated using CCK8 kit. The findings demonstrated that the OD450 value of the XMU-MP-1 treated group was substantially higher than that of the control group. Moreover, there was a dose-dependent increase in cell viability with increasing XMU-MP-1 concentration. Remarkably, EdU assay demonstrated enhanced proliferative capacity in the SW1783 (**Figure S1A**) and SW1088 (**Figure S1B**) cell lines treated with XMU-MP-1 concentration at 0.2% compared to normal medium. Likewise, colony Formation experiments have yielded consistent results (**Figures S1C, D**). The results indicate that XMU-MP-1 significantly enhances the proliferation of LGG cells. As the Hippo pathway has the potential to modulate the proliferation of t cells, we conducted an investigation into the impact of Hippo pathway-related gene (HPRGs) on prognosis at the bulk transcriptome level. Upon performing univariate Cox analysis on 19 Hippo/YAP pathway related genes in the literature, we were able to identify 16 HPRGs that exhibited significant prognostic value in meta cohort (**Figure 1D**).

This finding highlights the importance of Hippo/YAP signaling pathway in predicting the prognosis of patients with LGG.

### Identification of different activated states of the Hippo/YAP pathway in LGG cohorts

**Figure 2:**
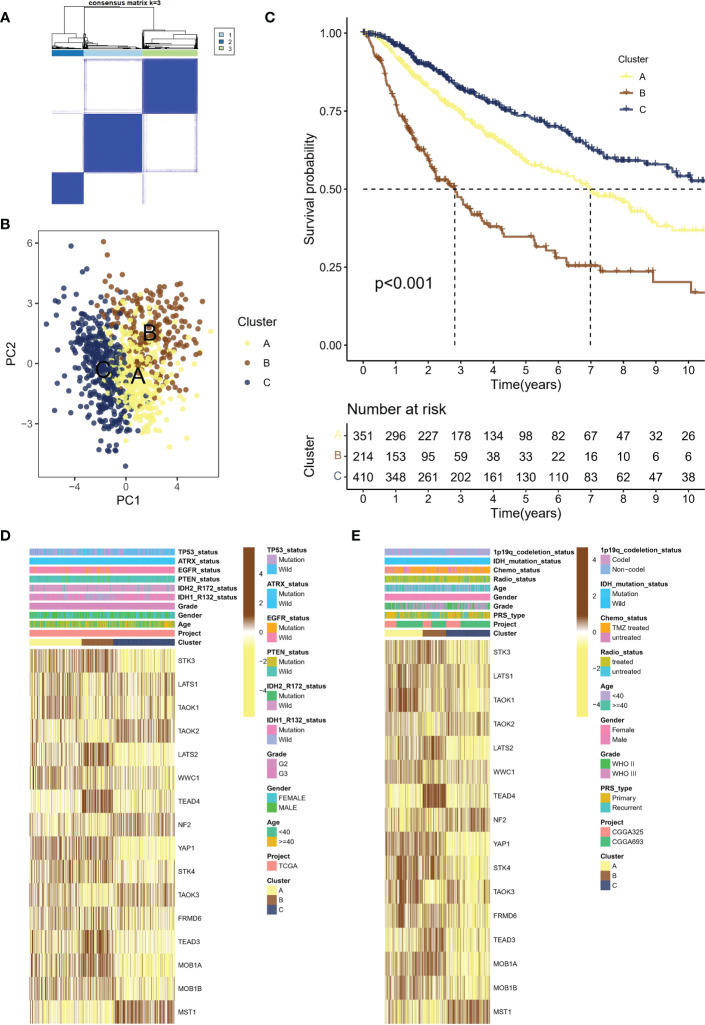
Molecular subtypes of LGG patients associated with Hippo/YAP pathway activation and their clinical outcomes. **(A)** Consensus clustering algorithm with k=3 classification of the meta cohort into three molecular subtypes. **(B)** Principal component analysis (PCA) of the three subtypes, showing significant heterogeneity and discreteness. **(C)** Kaplan-Meier survival analysis of the three subtypes, with subtype B having the worst prognosis. **(D)** Heat map showing the expression levels of HPRGs and clinicopathological factors in the TCGA cohort. **(E)** Heat map showing the expression levels of HPRGs and clinicopathological factors in the CGGA cohorts. LGG, lower-grade glioma; TCGA, The Cancer Genome Atlas; CGGA, Chinese Glioma Genome Atlas.

Using the consensus clustering algorithm with k=3 (**Figure 2A**), the meta cohort was classified into three molecular subtypes associated with Hippo/YAP Pathway activation profiles: A (n=351), B (n=214), and C (n=410). According to PCA (**Figure 2B**), the subtypes were significantly heterogeneous and discrete from each other. **Figure 2C** demonstrates that subtype B had the worst prognosis, subtype C had the best prognosis, and subtype A was in between. Heat mapping of clinicopathological factors and HPRGs was performed for subtypes in LGG patients. Genes such as STK3, LATS2, and TEAD4 were significantly activated in the poor prognosis B subtype in the TCGA-LGG cohort (**Figure 2D**), and similar results were observed in the CGGA cohort (**Figure 2E**). These analysis results, including the Kaplan-Meier survival curves, consistently demonstrated the same patterns of gene expression changes with the LGG prognosis, which enhances the robustness and reliability of our findings

These results support the theory that different subtypes based on HPRGs profiles are associated with different prognostic and clinical features. To be specific, subtype B had the worst prognosis, subtype C had the best prognosis.

### Tumor microenvironment variances among Hippo/YAP pathway activation states

**Figure 3:**
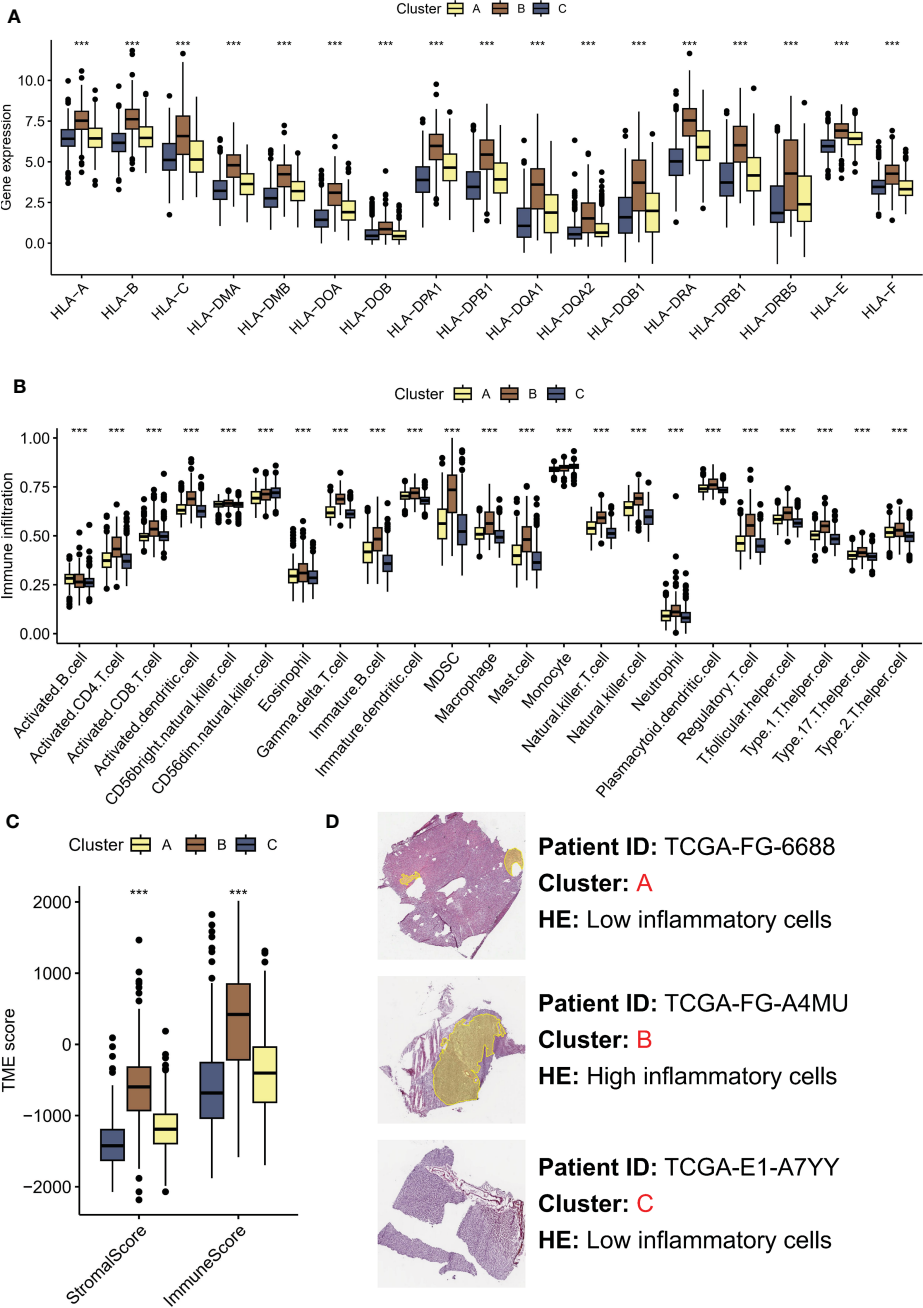
TME differences contribute to Hippo/YAP activation and subsequent cancer development. **(A)** HLA-related molecules expression in three molecular subtypes associated with Hippo/YAP pathway activation profiles. **(B)** Immune infiltration of three subtypes based on ssGSEA scores. **(C)** TME scores of three subtypes determined by the ESTIMATE algorithm. **(D)** HE images of the tumor microenvironment of three subtypes. ***P < 0.001. HLA: human leukocyte antigen; TME: tumor microenvironment; LGG: low-grade glioma; ssGSEA: single-sample gene set enrichment analysis; ESTIMATE: Estimation of STromal and Immune cells in MAlignant Tumors using Expression data.

Recent studies have highlighted the importance of the tumor microenvironment (TME) in shaping cancer progression, with evidence suggesting that TME variances may affect the activation status of the Hippo/YAP pathway (21). Understanding how TME differences contribute to Hippo/YAP activation and subsequent cancer development could provide new insights into the mechanisms of LGG growth and metastasis. The human leukocyte antigen (HLA) system plays a crucial role in the recognition and presentation of antigens by the immune system. Significantly elevated expression of HLA-related molecules was observed in subtype B, which was associated with a poor prognosis (**Figure 3A**). In greater detail, subtype B was observed to exhibit a high enrichment of immune cells according to ssGSEA. Specifically, the immune scores of subtype B were dominated by MDSC and Treg cells, which are known to have immunosuppressive effects (**Figure 3B**). Moreover, we employed the ESTIMATE algorithm to assess the TME landscape of various samples, which yielded consistent outcomes (**Figure 3C**). Nonetheless, it is noteworthy that the aforementioned MDSC and other cells may lead to immune evasion. Histopathological examination (HE) is a conventional method for distinguishing fibroblasts from inflammatory cells based on differences in cell and nucleus morphology and size. This approach enables identification of the distinct cell types present within a tissue sample and provides important insights into the cellular composition of the tumor microenvironment. As anticipated, subtype A exhibited increased infiltration of inflammatory cells (**Figure 3D**).

These results highlight the significance of TME variances in modulating cancer progression and their association with Hippo/YAP activation.

### Pathway enrichment analysis identifies suppressed Hippo/YAP pathway activity in subtype B with worst prognosis

**Figure 4:**
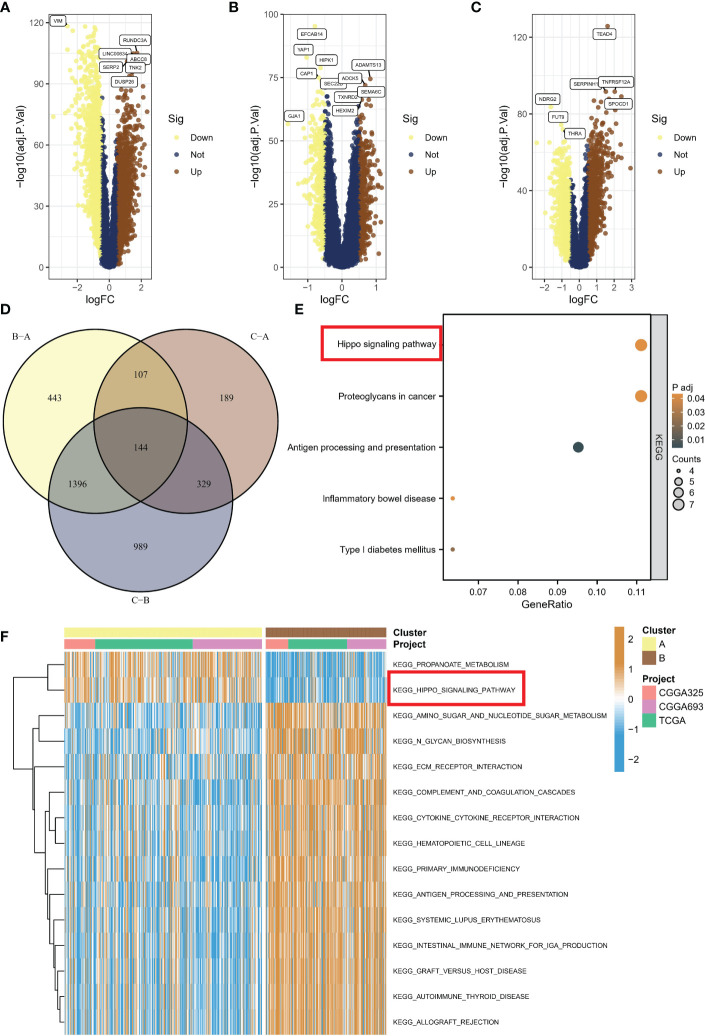
Activation of representative pathways across different subtypes. **(A)** Volcano plot of differentially expressed genes between subtype C and subtype B. **(B)** Volcano plot of differentially expressed genes between subtype C and subtype A. **(C)** Volcano plot of differentially expressed genes between subtype A and subtype B. **(D)** Overlap of dysregulated genes among different subtypes. **(E)** KEGG enrichment analysis of common genes. **(F)** GSVA showing decreased propanoate metabolic activity and suppressed Hippo pathway signaling in subtype B with poor prognosis. KEGG, Kyoto Encyclopedia of Genes and Genomes; GSVA, gene set variation analysis.

To investigate the activation of representative pathways across different subtypes, we analyzed differential gene expression. A total of 2855 genes were found to be dysregulated in subtype C compared to subtype B (**Figure 4A**), while 769 genes were dysregulated in subtype C compared to subtype A (**Figure 4B**), and 2090 genes were dysregulated in subtype A compared to subtype B (**Figure 4C**). Eventually, we identified 144 common genes after overlapping the above genes (**Figure 4D**). Upon conducting KEGG enrichment analysis of 114 genes, the Hippo/YAP pathway was unsurprisingly found to be the most significant item (**Figure 4E**). The GSVA indicated that subtypes B with a poor prognosis exhibited decreased propanoate metabolic activity and suppressed Hippo pathway signaling (**Figure 4F**).

In this section, we investigated pathway activation across different subtypes through differential gene expression analysis, identifying a total of 2855, 769, and 2090 dysregulated genes, with 144 common genes overlapping, and KEGG analysis indicating the Hippo/YAP pathway as the most significant; furthermore, GSVA revealed that subtypes B with poor prognosis displayed decreased propanoate metabolism and suppressed Hippo pathway signaling.

### Immune and targeted therapeutic efficacy is enhanced in subtype B

**Figure 5:**
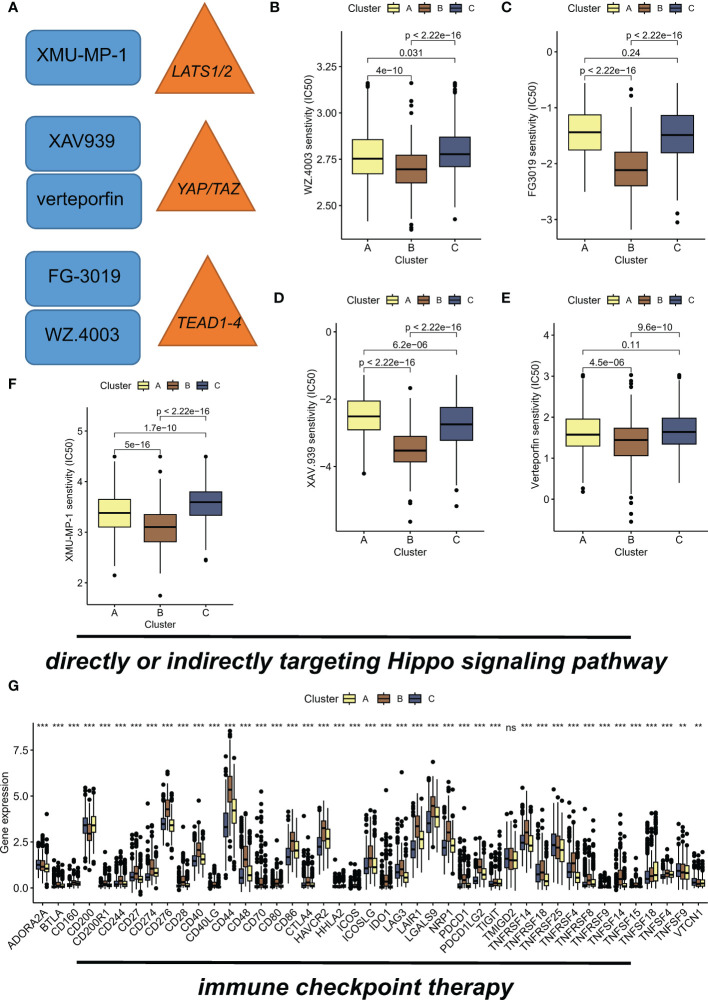
Hippo/YAP pathway-targeted drugs exhibit efficacy in suboptimal prognosis ubtype B. **(A)** Schematic diagram illustrating the Hippo/YAP pathway and its inhibitors. **(B)** Sensitivity of subtype B to WZ.4003, a small molecule inhibitor that targets the transcriptional co-activator YAP. IC50 values are presented for each subtype, with subtype B exhibiting the lowest IC50 value. **(C)** Sensitivity of subtype B to FG-3019, a monoclonal antibody that targets the ECM protein CTGF, a downstream effector of the Hippo pathway. IC50 values are presented for each subtype. **(D)** Sensitivity of subtype B to XAV.939, a tankyrase inhibitor that stabilizes the transcriptional co-activator protein YAP/TAZ, leading to enhanced Hippo pathway signaling and subsequent tumor suppression. IC50 values are presented for each subtype. **(E)** Sensitivity of subtype B to verteporfin, which inhibits the interaction between YAP and TEAD transcription factors. IC50 values are presented for each subtype. **(F)** Sensitivity of subtype B to XMU-MP-1, showing a dose-dependent increase in cell proliferation. IC50 values are presented for each subtype. **(G)** Elevated expression levels of immune checkpoints suggest that subtype B may respond favorably to immune checkpoint inhibitor (ICI) therapy. **P < 0.01; ***P < 0.001, ns indicates no statistical significance.

Recent studies have shown that inhibition of the Hippo/YAP pathway (**Figure 5A**) can sensitize cancer cells to chemotherapy and radiotherapy, indicating that targeting this pathway could enhance the efficacy of these treatments (23, 24). Hence, we aimed to investigate the potential of the different subtypes to guide therapeutic interventions in this section. WZ.4003 is a small molecule inhibitor that targets the oncogenic transcriptional co-activator YAP (25). FG-3019 is a monoclonal antibody that targets the ECM protein, connective tissue growth factor (CTGF), which is a downstream effector of the Hippo pathway (26). Inhibition of tankyrase by XAV.939 stabilizes the transcriptional co-activator protein YAP/TAZ, leading to enhanced Hippo pathway signaling and subsequent tumor suppression (27). Specifically, verteporfin inhibits the interaction between YAP and TEAD transcription factors. Of concern, suboptimal prognosis subtype B exhibited sensitivity to drugs WZ.4003 (**Figure 5B**), FG-3019 (**Figure 5C**), XAV.939 (**Figure 5D**), verteporfin (**Figure 5E**), and XMU-MP-1 (**Figure 5F**). Specifically, subtype B had the lowest IC50 value compared to the other subtypes. Moreover, the elevated expression levels of numerous immune checkpoints suggest that subtype B may respond favorably to immune checkpoint inhibitor (ICI) therapy (**Figure 5G**).

### Development of an integrated Hippo/YAP pathway-related signature

**Figure 6:**
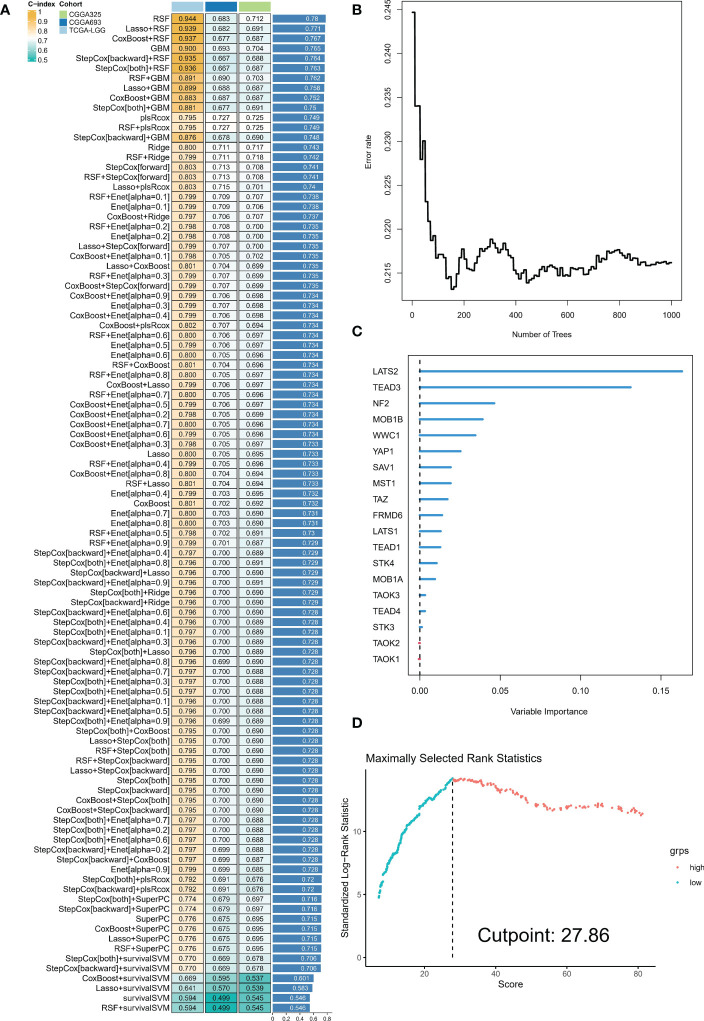
Composite machine learning model predicts survival risk profiles and Hippo/YAP pathway status in LGG patients. **(A)** Comparison of C-index values for various machine learning models tested. Random forest tree model showed the highest C-index and was thus chosen as the best model. **(B)** Plot of error rate versus number of trees grown in the random forest model. The error rate remained stable as the number of trees increased, indicating robust performance of the model. **(C)** Sequence of importance of 19 Hippo pathway-related genes (HPRGs) in the random forest model. **(D)** Optimal cutoff value of 27.86 was determined for identifying patients with high and low survival risk.

**Figure 7:**
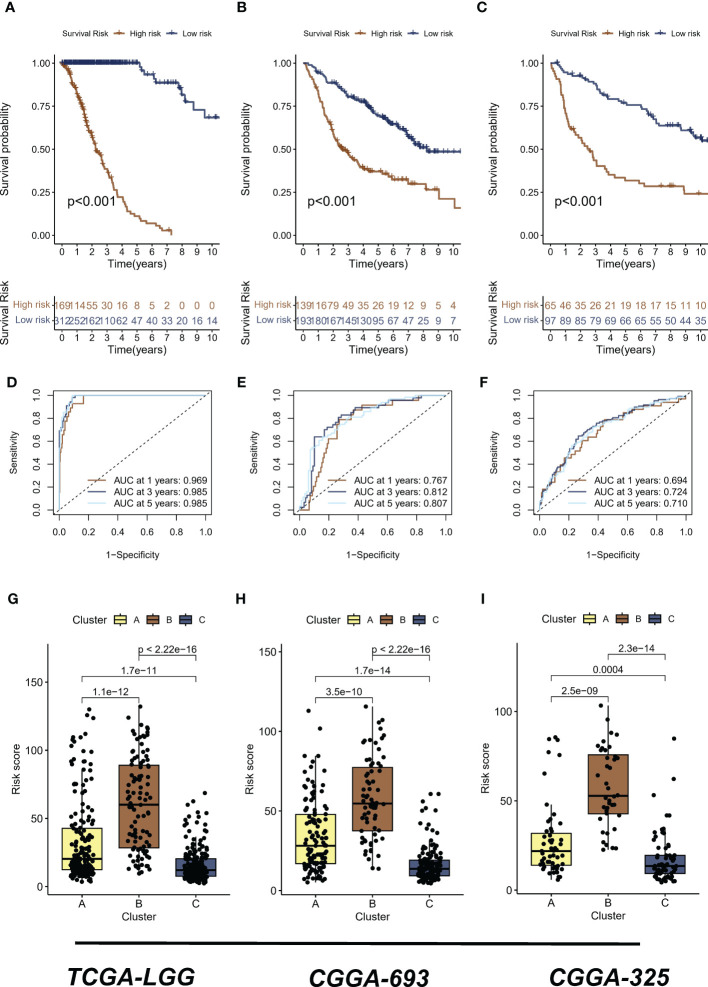
Risk stratification and predictive accuracy in LGG cohorts. **(A-C)** Kaplan-Meier curves were plotted for overall survival (OS) in low-risk and high-risk groups of TCGA-LGG, CGGA-325, and CGGA-693 cohorts, respectively. **(D)** The area under the receiver operating characteristic curve (AUC) for 1-, 3-, and 5-year OS in the TCGA-LGG cohort. **(E)** The area under the receiver operating characteristic curve (AUC) for 1-, 3-, and 5-year OS in the CGGA-693 cohort. **(F)** The area under the receiver operating characteristic curve (AUC) for 1-, 3-, and 5-year OS in the CGGA-325 cohort **(G–I)** The distribution of high risk scores, primarily concentrated in subtype B, was observed in TCGA-LGG, CGGA-325, and CGGA-693 cohorts, respectively. LGG, lower-grade glioma; TCGA, The Cancer Genome Atlas; CGGA, Chinese Glioma Genome Atlas.

The analyses presented above were conducted on a patient cohort with LGG. To predict individual patients with different survival risk profiles, we used a composite machine learning model. Moreover, this model predicted status of the Hippo/YAP pathway in these patients. Among the various models we tested, the random forest tree model had the highest C-index and was thus determined to be the best model (**Figure 6A**). As training progressed, the error rate remained stable (**Figure 6B**). The rank of importance of 19 HPRGs was displayed, and LATS2 was found to be the most important gene in the model (**Figure 6C**). Using the internal risk score of the model, we determined 27.86 as the optimal cutoff value for identifying patients with high and low survival risk (**Figure 6D**). Risk stratification was carried out on three cohorts, TCGA-LGG, CGGA-325, and CGGA-693, using consistent cut-off values. The Kaplan-Meier curves demonstrated that the low-risk group exhibited significantly prolonged overall survival in the TCGA-LGG cohort (**Figure 7A**). Similarly, this trend was observed in both the CGGA-693 (**Figure 7B**) and CGGA-325 (**Figure 7C**) testing cohorts. In the TCGA-LGG cohort (**Figure 7D**), the area under the receiver operating characteristic curve (AUC) for 1-, 3-, and 5-year overall survival (OS) were all greater than 0.9, indicating high predictive accuracy. Similar findings were observed in the test set, with AUC values greater than or near 0.7, particularly around 0.8 in the CGGA-693 cohort (**Figures 7E, F**). We have successfully identified individual patients with high-risk scores, which were found to be primarily concentrated in subtype B, in both the training and validation sets, confirming our initial suspicions (**Figures 7G–I**).

## Discussion

Our study aimed to investigate the potential use of the Hippo/YAP pathway in predicting prognosis, immune microenvironment, and therapeutic regimen in patients with LGG. The results of the study showed that the activation of the Hippo/YAP pathway was associated with different prognostic and clinical features, and could be used to guide personalized treatment strategies for LGG. The use of machine learning models in many aspects has been gaining increasing attention in recent years (28–30). We used a composite machine learning model to predict individual patients’ survival risk profiles and the Hippo/YAP pathway status. The results of the model showed that the Hippo/YAP pathway status could be used as a predictor of patient survival in LGG. This is an important finding, as predicting patient survival is crucial for developing effective treatment strategies.

We also investigated the efficacy of small molecule inhibitors targeting the Hippo/YAP pathway. The results of the study showed that the XMU-MP-1 inhibitor significantly enhanced the proliferation of LGG cells, indicating that targeting the Hippo/YAP pathway could be an effective therapeutic strategy for LGG. The small molecular compound XMU-MP-1 is a potent and selective inhibitor of MST1/2 (22). While inhibition of Hippo signaling may increase proliferation in LGG cells, it is also possible that it could lead to apoptosis or senescence, which could limit tumor growth (31). More studies are needed to fully understand the therapeutic potential of Hippo pathway inhibition in LGG. Similarity, the restoration of cell viability and reversal of HHT-induced hepatocellular carcinoma apoptosis were observed following treatment with XMU-MP-1 (32). Chao et al. (33) reported that MST1 down-regulation promoted glioma cell proliferation and growth, while inhibiting apoptosis. Conversely, MST1 overexpression inhibited glioma cell proliferation by modulating the AKT/mTOR pathway. Our finding is supported by previous studies, which have shown that targeting the Hippo/YAP pathway can suppress tumor growth and invasion in various types of cancer (31).

Consensus clustering algorithm was used to classify the meta cohort into three molecular subtypes associated with Hippo/YAP Pathway-related profiles. The results of the study showed that different Hippo/YAP Pathway activation profiles were associated with different prognostic and clinical features. Subtype B, for example, had the lowest IC50 value, indicating sensitivity to drugs that target the Hippo/YAP pathway. This suggests that patients with subtype B LGG may benefit from treatment with Hippo/YAP pathway inhibitors. Furthermore, GSVA revealed that subtypes B with poor prognosis displayed decreased propanoate metabolism and suppressed Hippo pathway signaling. The Hippo signaling pathway is highly conserved from *Drosophila melanogaster* to mammals and plays a crucial role in organ size control, tissue regeneration, and tumor suppression. When the pathway is activated, resulting in YAP/TAZ retention (inactivity) and degradation in the cytoplasm (34). Therefore, our study suggests that targeting individuals with low YAP expression and activating the Hippo pathway could be a crucial step towards personalized therapy. Verteporfin, a drug that has been approved by the FDA for use in photodynamic therapy for macular degeneration, has been shown to inhibit YAP (35). Despite its effectiveness as an inhibitor, verteporfin has yet to be approved for clinical tumor treatment due to its unclear function.

In addition, the study also investigated the immune microenvironment of LGG. The immune scores of subtype B were dominated by MDSC and Treg cells, which are known to have immunosuppressive effects. Interestingly, using a murine prostate adenocarcinoma model, Wang et al. (36) demonstrated that cancer cells producing CXCL5 under the control of YAP can recruit MDSCs to the tumor site via heterotypic CXCL5 binding to CXCR2 receptors. Moreover, YAP was discovered to function downstream of the PRKCI oncogene to upregulate TNFα expression, recruit MDSCs, and inhibit the function of cytotoxic T cells (CTLs) (37). Proposals have been made by various groups that suggest PD-L1 can promote chemotherapy resistance, metastasis, and also signal within cancer cells to evade cytotoxicity (38, 39). It is surprising that PD-L1 can upregulate YAP expression levels in NSCLC lines. Recently, Tung et al. reported that increased generation of reactive oxygen species (ROS) associated with PD-L1 expression in NSCLC lines led to upregulation of hypoxia-inducible factor 1α (HIF1α) (40). This finding suggests that the Hippo/YAP pathway may play a role in regulating the immune microenvironment of LGG, and targeting this pathway may enhance the efficacy of immunotherapy for LGG.

Despite the promising findings of this study, there are several limitations that must be acknowledged. In the methodology, we only used one model for clustering, which is principal component analysis (PCA). However, it would be useful to cross-validate our results using different clustering methods in the future. First, the *in vitro* models used in this study may not completely reflect the complexity of tumor microenvironments and the heterogeneity of patient populations, which may limit the generalizability of the results. Second, although a comprehensive list of Hippo/YAP pathway-related genes was used in this study, it is possible that other genes or pathways may also contribute to the development and progression of LGG. Third, the sample size of this study is relatively small, and larger studies are needed to validate the findings and improve the accuracy of the machine learning model. Finally, this study only focused on the Hippo/YAP pathway, and further studies are needed to investigate the potential interactions between this pathway and other signaling pathways involved in LGG.

## Conclusions

In conclusion, this study demonstrates the potential use of the Hippo/YAP pathway in predicting the prognosis, immune microenvironment, and therapeutic regimen of patients with LGG. The different Hippo/YAP Pathway activation profiles associated with different prognostic and clinical features suggest the potential for personalized treatments. Further studies are needed to validate the findings of this study and to develop effective therapeutic strategies targeting the Hippo/YAP pathway in LGG.

## Data availability statement

The original contributions presented in the study are included in the article/**Supplementary Material**. Further inquiries can be directed to the corresponding author.

## Author contributions

Y-DG and JS conceived and designed the study. CZ was responsible for materials. LH drafted the article. C-JY and H-WZ revised the article critically. All authors had final approval of the submitted versions.

## Supplementary material

Supplementary Figure 1 Edu and colony formation. (A) Edu assay treated with different media in SW1783. (B) Edu assay treated with different media in SW1088. (C) Colony formation treated with different media in SW1783. (D) Colony formation assay treated with different media in SW1088.
