# Supplementary figures and images for: Comprehensive analysis and molecular map of Hippo signaling pathway in lower grade glioma: the perspective toward immune microenvironment and prognosis
Source: Front Oncol. 2023 May 12;13:1198414. doi: 10.3389/fonc.2023.1198414 (PMC10213431; doi:10.3389/fonc.2023.1198414)

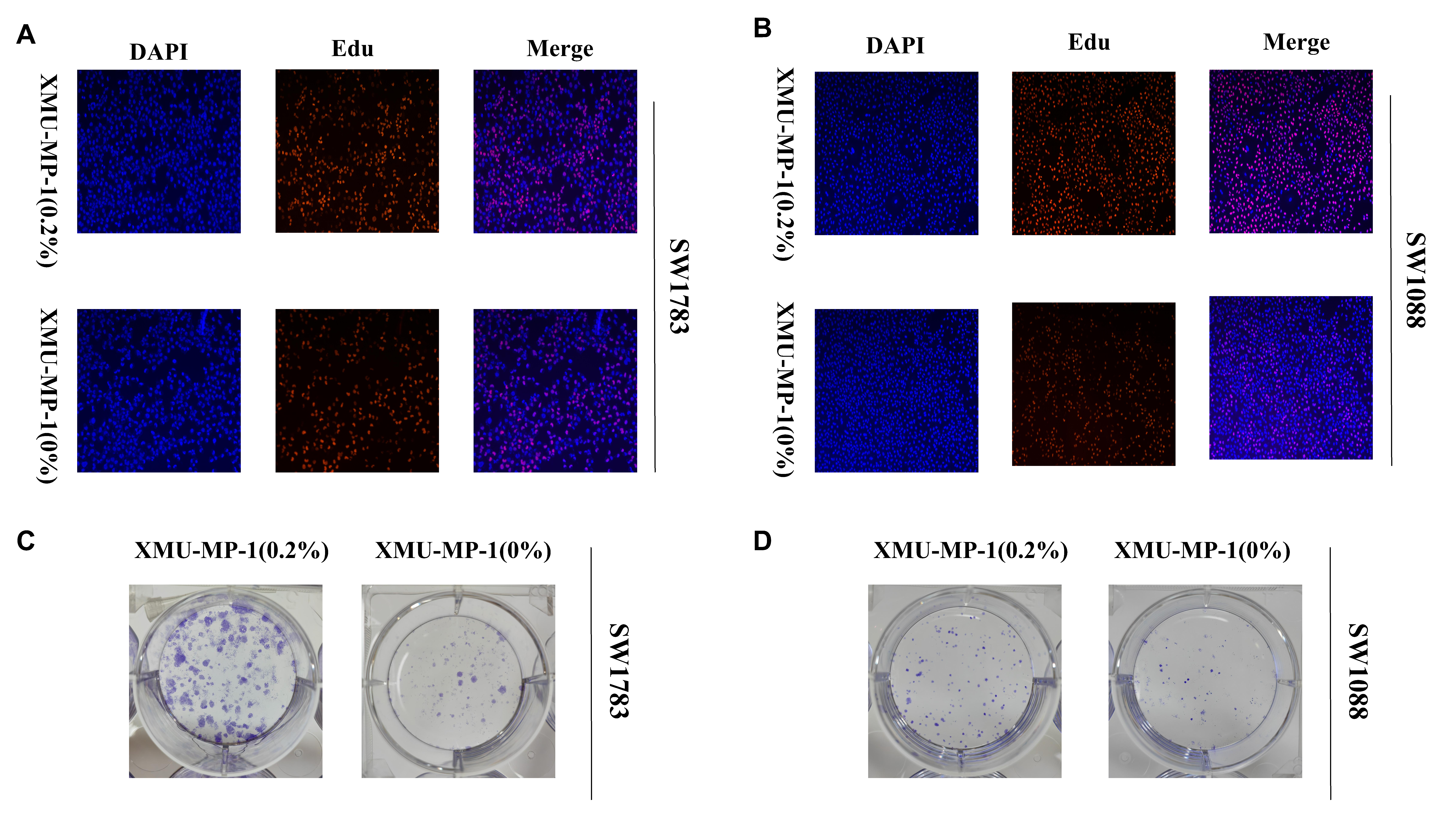

Supplement: Supplementary Figure 1 — Edu and colony formation. (A) Edu assay treated with different media in SW1783. (B) Edu assay treated with different media in SW1088. (C) Colony formation treated with different media in SW1783. (D) Colony formation assay treated with different media in SW1088. [file Image_1.tif]
